# Structural and photoluminescence studies on catalytic growth of silicon/zinc oxide heterostructure nanowires

**DOI:** 10.1186/1556-276X-8-174

**Published:** 2013-04-17

**Authors:** Su Kong Chong, Chang Fu Dee, Saadah Abdul Rahman

**Affiliations:** 1 Low Dimensional Materials Research Centre, Department of Physics, University of Malaya, Kuala Lumpur 50603, Malaysia; 2 Institute of Microengineering and Nanoelectronics (IMEN), Universiti Kebangsaan Malaysia (UKM), Bangi, Selangor 43600, Malaysia

**Keywords:** Silicon, Zinc oxide, Core-shell, Hierarchical, Nanowires, Photoluminescence

## Abstract

Silicon/zinc oxide (Si/ZnO) core-shell nanowires (NWs) were prepared on a p-type Si(111) substrate using a two-step growth process. First, indium seed-coated Si NWs (In/Si NWs) were synthesized using a plasma-assisted hot-wire chemical vapor deposition technique. This was then followed by the growth of a ZnO nanostructure shell layer using a vapor transport and condensation method. By varying the ZnO growth time from 0.5 to 2 h, different morphologies of ZnO nanostructures, such as ZnO nanoparticles, ZnO shell layer, and ZnO nanorods were grown on the In/Si NWs. The In seeds were believed to act as centers to attract the ZnO molecule vapors, further inducing the lateral growth of ZnO nanorods from the Si/ZnO core-shell NWs via a vapor-liquid-solid mechanism. The ZnO nanorods had a tendency to grow in the direction of [0001] as indicated by X-ray diffraction and high resolution transmission electron microscopy analyses. We showed that the Si/ZnO core-shell NWs exhibit a broad visible emission ranging from 400 to 750 nm due to the combination of emissions from oxygen vacancies in ZnO and In_2_O_3_ structures and nanocrystallite Si on the Si NWs. The hierarchical growth of straight ZnO nanorods on the core-shell NWs eventually reduced the defect (green) emission and enhanced the near band edge (ultraviolet) emission of the ZnO.

## Background

One-dimensional semiconductor nanowires (NWs) have gained tremendous attention owing to their unique optical and electrical properties, which can be applied in nanophotonics and nanoelectronics [1,2]. Among the semiconductor NWs, silicon (Si) and zinc oxide (ZnO) NWs are leading in numerous energy-related applications, especially in the fields of optics [3,4], photovoltaic [5,6], and field emission [7,8]. Si exhibits an indirect band gap of 1.12 eV, which prevents it from emitting visible light. However, nanocrystalline Si as well as Si NWs can produce red emission due to the quantum confinement effect [9,10]. This makes them applicable in photonics [3]. ZnO nanorods (NRs) are also known to exhibit efficient ultraviolet (UV) and visible green emissions at room temperature [11]. The UV emission is attributed to the near band edge emission of ZnO [12,13] (E_g_ approximately 3.37 eV), while the green emission is generally known to be a defect emission due to oxygen vacancies or oxide antisite in ZnO NRs [14-16].

The combination of Si NWs and ZnO nanostructures to form nanoparticle (NP)-decorated core-shell and branched hierarchical NWs could significantly improve the shortcomings of each individual Si or ZnO nanostructures. One interesting approach is to obtain white emission by combining the different emission regions of both Si and ZnO nanostructures. A flat and broad range of visible light emission ranging from approximately 450 to 800 nm were independently demonstrated using a porous Si/ZnO core-shell NWs [17] and ZnO/amorphous Si core-shell NWs [18]. Meanwhile, tunable photoluminescence (PL) from visible green to UV emission can be achieved by varying the thickness of SiO_2_ layer for ZnO/SiO_2_ core-shell NRs [19]. Another example is the enhancement of the electron field emission properties, where an extremely low turn-on field <1 V/μm and field enhancement factor of approximately 10^4^ were obtained from an ultrathin ZnO film (approximately 9 nm) coated Si nanopillar arrays [20]. Similar field enhancement results were also obtained by several groups using ZnO NP-decorated Si NWs [21] and ZnO NWs/Si nanoporous pillar arrays [22].

To date, there are several studies using different techniques in regards to the synthesis of the heterostructured Si/ZnO core-shell NWs and hierarchical NWs [17,20-27]. In general, the growth of Si NWs core and ZnO nanostructures shell was carried out by means of a two-step deposition. Most of the studies focused on the top-down method to fabricate Si NW arrays via a dry reactive etching [20,23] and a wet metal-assisted etching [17,21,22,24-27] techniques. It is important to note that this method of producing Si NWs is usually accompanied by surface defects and impurity issues [28,29]. The Si/ZnO core-shell NWs can be formed by the settling of a ZnO layer on the Si NWs using atomic layer deposition [20,21,24], pulsed laser deposition [23], or metal-organic chemical vapor deposition [17]. Meanwhile, three-dimensional hierarchical NWs with Si wire/NWs core and ZnO NRs branches were successfully grown using vapor transport and condensation [22,25] or hydrothermal growth [26,27] methods. In order to form the hierarchical heterostructured NWs, the interspacing between Si NW cores must be large enough (in other words, the density of Si NWs on the substrate must be low enough) to provide enough space for the lateral growth of ZnO NRs from the Si NWs. In this particular case, chemical vapor deposition method is a better approach to obtain the Si NWs array due to its capability of producing NWs with lower density and larger gaps compared to the metal-assisted etching method [30].

In this work, we present a study on the growth of ZnO nanostructures on Si NWs using an In catalyst. Tapered Si NW arrays were first synthesized by following a vapor-liquid-solid (VLS) mechanism using In catalyst and a hot-wire chemical vapor deposition [31]. In seeds were then coated on the as-grown Si NWs using the same system. This was followed by the synthesis of ZnO nanostructures using vapor transport and condensation. The method was carried out by way of a thermal evaporation of graphite-mixed ZnO powder [32]. The ZnO nanostructures formed at different growth time were then studied. Structural, compositional, and optical properties of the as-grown samples were characterized using field emission scanning electron microscopy (FESEM), high-resolution transmission electron microscopy (HRTEM), energy dispersive X-ray (EDX), X-ray diffraction (XRD), and PL spectroscopy methods.

## Methods

Si NWs were synthesized on a p-type Si(111) substrate using a home-built plasma-assisted hot-wire chemical vapor deposition system [33]. In catalysts with sizes ranging from 40 to 100 nm were coated on the substrate prior to the synthesis of Si NWs. Silane gas diluted in hydrogen (H_2_) gas in a ratio of 1:20 (5:100 sccm) was used as the Si source for the growth of Si NWs. The details of the deposition process and parameters have been previously described [31,34-37]. The as-grown Si NWs were first coated with a layer of In seeds using the same system. Next, 1.3 ± 0.1 mg of In wire was hung on a tungsten filament 3 cm above the Si NWs substrate. The In wire was evaporated at filament temperature of approximately 1,200°C under a hydrogen plasma environment to produce nano-sized In seeds [31]. The H_2_ flow rate and rf power of the plasma were fixed at 100 sccm and 40 W, respectively. The In seed-coated Si NWs (In/Si NWs) substrate was then transferred into a quartz tube furnace for the ZnO nanostructures deposition.

ZnO nanostructures were deposited onto the In/Si NWs via a vapor transport and condensation process. A mixture of ZnO and graphite (1:1) powders with a total weight of approximately 0.2 g was placed at the hot zone center of the quartz tube. One end of the quartz tube was sealed and connected to N_2_ gas inlet, while the other end remained open. The In/Si NWs substrate was then inserted through the open end and placed at approximately 12 cm from the evaporation source. The mixed powder was heated to approximately 1,100°C at different durations starting from 0.5 to 2 h. The sample substrates placed downstream of the quartz tube resulted in a gradient temperature change of 600 to 500°C from the center towards the opened end.

Morphologies of the samples were observed from a Hitachi SU 8000 FESEM (Chiyoda-ku, Japan). An EDAX Apollo XL SDD detector EDX spectroscopy (Mahwah, NJ, USA) attached to the FESEM was utilized for the composition analysis of the samples. TEM and HRTEM micrographs as well as the fast Fourier transform (FFT) electron diffraction patterns of the samples were studied using a JEOL JEM 2100F HRTEM (Akishima-shi, Japan). A SIEMENS D5000 X-ray diffractometer (Munich, Germany) was used to obtain the XRD pattern of the samples. The measurements were performed at a grazing angle of 5°. PL spectra were recorded using a Renishaw InVia PL/Raman spectrometer (Wotton-under-Edge, UK) under an excitation He-Cd laser source of 325 nm.

## Results and discussion

**Figure 1:**
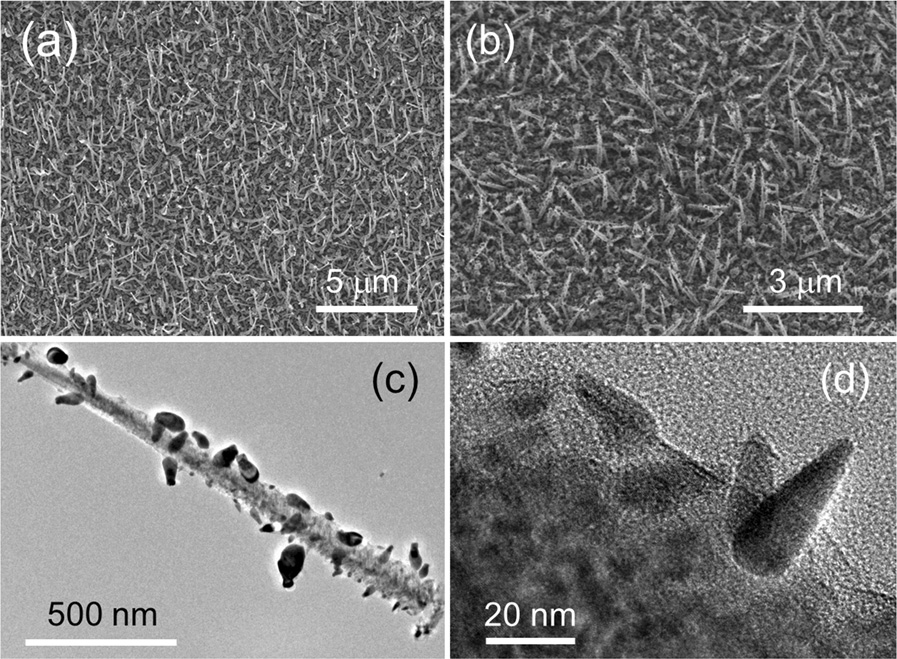
**SEM and TEM studies on the In/Si NWs.** FESEM images of (**a**) Si NWs and (**b**) In seeds coated on Si NWs. (**c**) TEM and (**d**) HRTEM micrographs of the In seeds coated on the surface of the Si NW.

Figure 1a shows the FESEM image of the as-grown In-catalyzed Si NWs. The NWs revealed tapered structures with average base and tip diameters of approximately 100 and 20 nm, respectively. The average length of the NWs is about 2 μm. In seeds coated on the Si NWs by evaporation are illustrated by FESEM as shown in Figure 1b. TEM (Figure 1c) and HRTEM (Figure 1d) micrographs reveal the cone-shaped In seeds with sizes varying from 8 to 50 nm, which are evenly distributed on the surface of the NWs. This adhesion of the In seeds on the Si NWs is confirmed by the HRTEM where the crystal lattices of both the In and Si crystals are observed in Figure 1d. The high sticky coefficient of In seeds [38] allows it to act as centers to collect vaporized ZnO molecules/atoms, which then nucleate to form ZnO nanostructures on the Si NWs.

**Figure 2:**
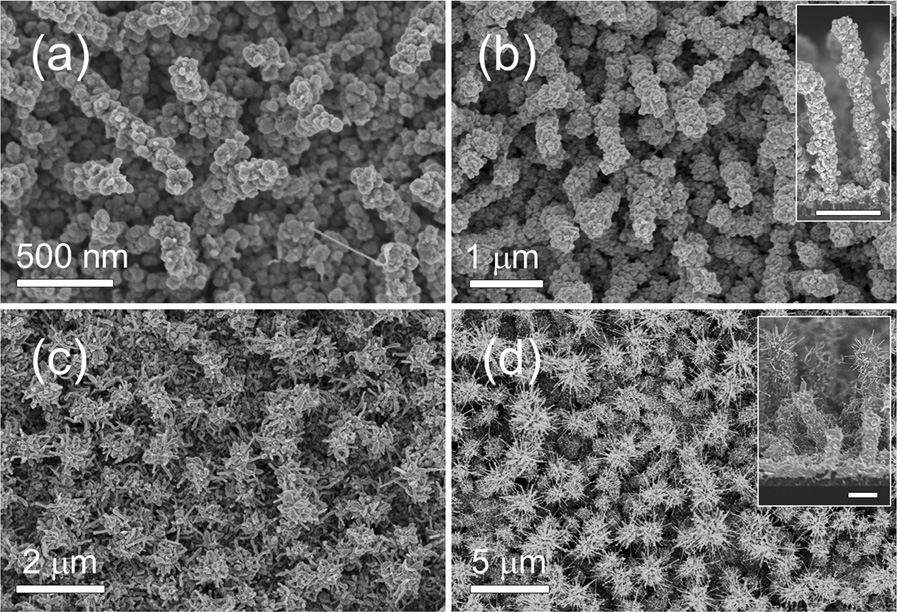
**Morphology study of the ZnO nanostructures grown on In/Si NWs.** FESEM images of ZnO nanostructures formed on In/Si NWs at different growth times of (**a**) 0.5, (**b**), (**c**) 1.5, and (**d**) 2 h. Insets in (**b**) and (**d**) are the cross-sectional images of the respective figures (scale bar = 1 μm).

Morphologies of the ZnO nanostructures grown on the In/Si NWs at different growth times between 0.5 to 2 h are displayed by the FESEM images in Figure 2a,b,c,d. In Figure 2a, high density of ZnO NPs is observed on the surface of the In/Si NWs. Upon further condensation of ZnO vapors, the ZnO NP-decorated structures were transformed into NPs shell layer cladding the surface of the NWs (Figure 2b). It is found that the average diameter of the NWs increased to approximately 200 ± 10 nm after 0.5 h and approximately 260 ± 20 nm after 1 h of ZnO vapors condensation. These Si/ZnO core-shell NWs exhibit a rough surface due to the ZnO NPs coating (inset in Figure 2b). Further increase in ZnO growth time to 1.5 h induced the growth of ZnO NRs from the In/Si NWs surface, resulting in the formation of Si/ZnO hierarchical core-shell NWs. The NRs with an average diameter 32 ± 10 nm and lengths varying from tens to approximately 500 nm are randomly elongated from the surface of the NWs. The length and average diameter of the NRs increase to approximately 400 nm to approximately 1 μm and 45 ± 13 nm, respectively, after 2 h of ZnO vapor condensation. The cross-sectional image (inset in Figure 2d) clearly shows that the ZnO NRs were hierarchically grown from the lateral surface of the Si NWs.

The initial growth stage of the ZnO NRs can be observed from the FESEM and TEM micrographs (Additional file 1: Figure S1). Catalyst particles can be clearly seen on the tip of the ZnO NRs (white circles in Additional file 1: Figure S1a). This suggests that a VLS growth mechanism was involved in the growth of ZnO NRs [39,40]. The observed large variation of the ZnO NR lengths (Figure 2c,d) is also indicative of a catalytic growth process for the ZnO NRs. Due to the different sizes of the In catalyst seeds, the nucleation time as well as the growth rate of the ZnO NRs can vary [41]. Thus, in this case, In seeds have two roles: first is to act as a center to attract vaporized molecules/atoms to form the ZnO shell layer covering the Si NWs, and second is to catalyze the growth of ZnO NRs when the amount of ZnO reaches a certain critical point. Similar to tin (Sn), In is one of the rare materials which forms alloy with Zn and exists at low eutectic temperature of approximately 150°C at 3% of Zn [42]. Several studies have revealed that Sn could catalyze the growth of ZnO NRs via a VLS growth mechanism [43,44]. Our results showed that In carried out the same role as well. A lattice-resolved HRTEM image was taken at the interface ZnO and In structures as shown in Additional file 1: Figure S2. In contrast to the single crystalline structure of ZnO NR, the In seed showed an amorphous structure. This could be due to the incorporation of oxygen and Zn elements into the In seeds, thus forming Zn-doped In_2_O_3_ structure during the ZnO deposition process [45].

**Figure 3:**
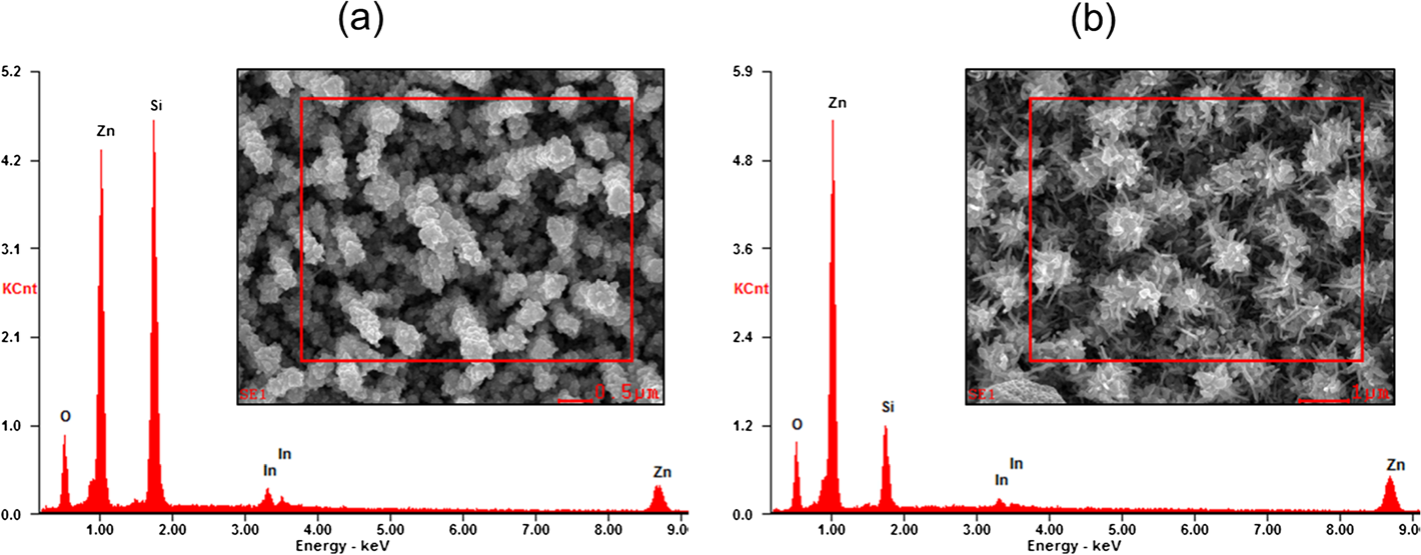
**EDX analysis on the Si/ZnO heterostructure NWs.** EDX spectra of the (**a**) Si/ZnO core-shell NWs and (**b**) Si/ZnO hierarchical core-shell NWs.

The composition of the ZnO nanostructures deposited on In/Si NWs is examined by EDX spectroscopy. The EDX spectra taken from the Si/ZnO core-shell and hierarchical core-shell NWs are shown in Figure 3a,b, respectively. Zn and O peaks are mainly from the shell layer of the NWs. We believed that the Si peak could have originated from the core of Si NWs and also from the Si substrate. On the other hand, the In signal originated from the In seeds which coated on the Si NWs surface. High signal level of Zn and O elements (Zn: O at % = 1.0:0.7) confirmed the coating of ZnO nanostructures on the Si NWs. The significant increase in the value of Zn peak, together with the suppression of Si peak (Figure 3b), may to some extent indicate the higher condensation of ZnO, forming laterally-grown ZnO NRs.

**Figure 4:**
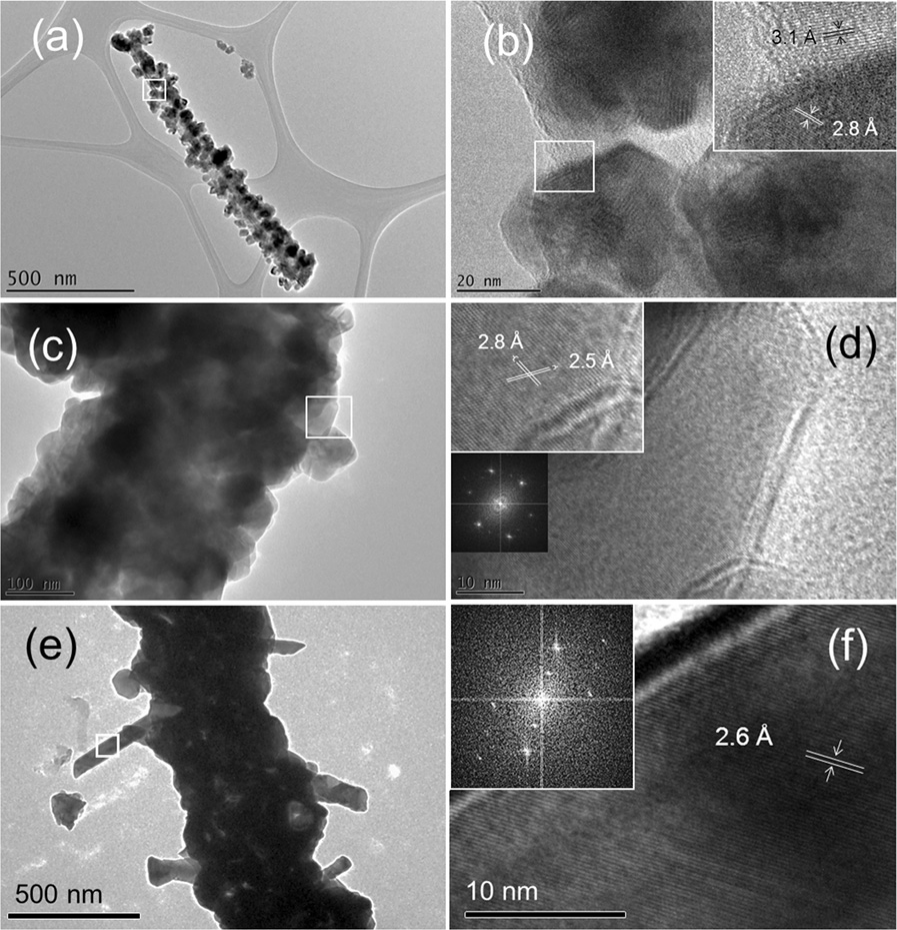
**HRTEM analysis on the Si/ZnO heterostructure NWs.** TEM and HRTEM micrographs of Si/ZnO core-shell NWs prepared at different ZnO growth time of (**a**, **b**) 0.5, (**c**, **d**) 1, and (**e**, **f**) 1.5 h. Magnified HRTEM micrographs from (**b**) and (**d**) are inserted in the respective figures. FFT patterns inserted in (**d**) and (**f**) are converted from the appropriate HRTEM micrographs.

The structures of the ZnO NPs and NRs layers grown on the In/Si NWs were characterized by HRTEM. Figure 4a shows a TEM micrograph of a ZnO NPs decorating NWs prepared at 0.5 h of ZnO deposition time. Hexagonal shaped ZnO NPs with different sizes from 10 to 40 nm were observed on the surface of the Si NWs. A magnified HRTEM micrograph of the open square area in Figure 4a is displayed in Figure 4b. A lattice-resolved HRTEM image (inset of Figure 4b) shows the crystal lattice at the interface of Si and ZnO structures. The estimated lattice spacing at two different locations for Si(111) and ZnO(100) crystallographic planes are 3.1 and 2.8 Å, respectively. The average sizes of ZnO NPs measured by the TEM system increased to approximately 60 ± 10 nm, which corresponds to the increase of the ZnO growth time to 1 h. The TEM micrograph (Figure 4c) shows the Si NWs are mostly covered by the ZnO NPs. The HRTEM micrograph (Figure 4d) shows the high crystallinity of the grown ZnO NPs. A set of measured lattice spacing with values of approximately 2.8 and 2.5 Å confirms to the ZnO(100) and (101) crystal planes given by the FFT pattern shown in the inset of Figure 4d. These crystal planes have also been reported by other researchers as a favorable orientation for ZnO NPs grown on Si NWs [17,21]. The Si/ZnO hierarchical core-shell NW consists of multiple ZnO NRs which grew laterally from the side of the Si/ZnO core-shell NWs, as revealed in Figure 4e. The lattice-resolved HRTEM image in Figure 4f shows a lattice spacing of approximately 2.6 Å which corresponds to ZnO(002) crystallographic plane. FFT pattern (inset of Figure 4f) indicates that the ZnO NRs are growing along the direction of [0001]. This corresponds with the observation of the growth direction for branching ZnO NRs on the Si wire [27] and undoped ZnO cores previously reported [46].

**Figure 5:**
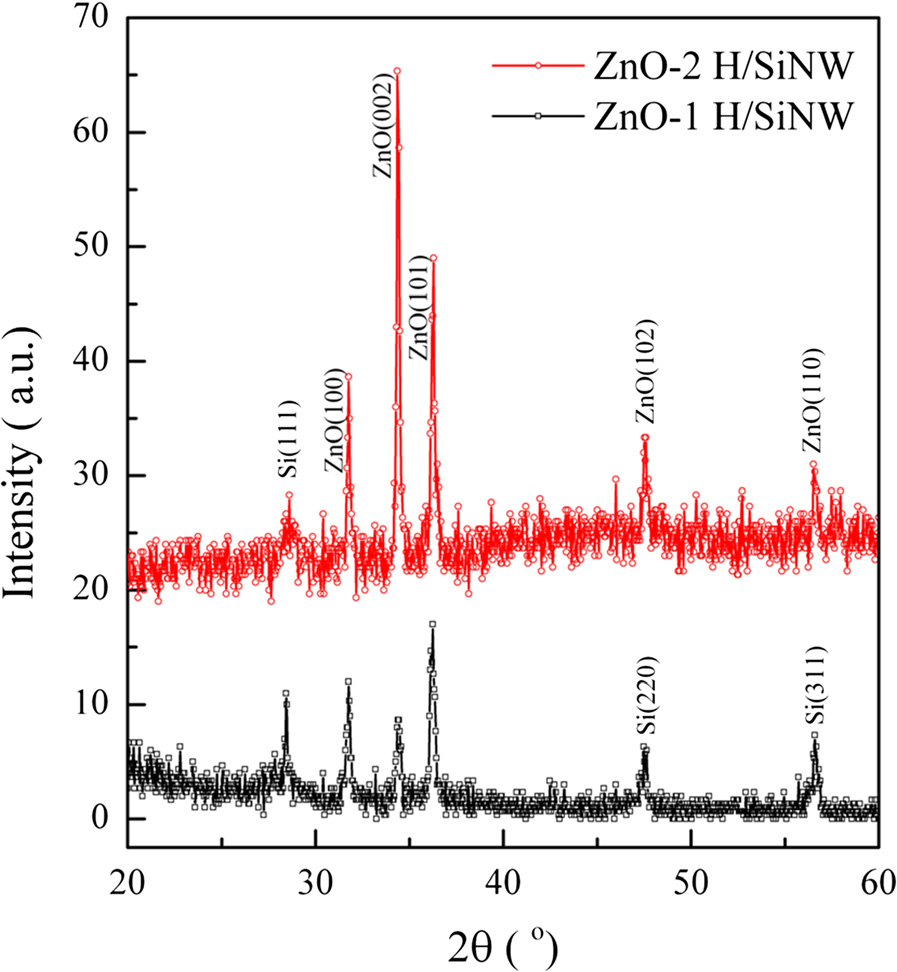
**XRD study on the Si/ZnO heterostructure NWs.** XRD pattern of the ZnO nanostructures prepared at ZnO growth time of 1 and 2 h on the In/Si NWs.

The crystal structures of the samples were studied using XRD. Figure 5 shows the XRD pattern of the Si/ZnO core-shell NWs prepared at the ZnO growth duration of 1 and 2 h. The Si diffraction peaks are indexed to a face-centered cubic structure [31], while ZnO diffraction peaks are matched to the structure of wurtzite (JCPDS card: 36–1451). The XRD pattern for ZnO nanostructures formed on Si NWs at ZnO growth time of 1 h revealed a similar structure as bulk ZnO [47] with the strongest diffraction peak being at ZnO(101) crystal plane. Parallel to the observation of FESEM and TEM images, the XRD pattern indicates that the in-plane growth is more dominant compared to the one-dimensional growth of ZnO at a growth time of 1 h. The intensity of ZnO crystal peaks increased with the rise in ZnO growth time to 2 h. In addition, the ZnO(002) crystalline peak became more prevalent with longer ZnO growth time. The strong ZnO(002) peak proves the *c*-axis growth of ZnO along the [0001] growth direction. This again shows that prolonging the growth time will switch the deposition of ZnO materials from solely expanding the thickness of the shell layer to lateral growth of ZnO NRs out of the Si/ZnO radial which gives a stronger ZnO(002) peak.

**Figure 6:**
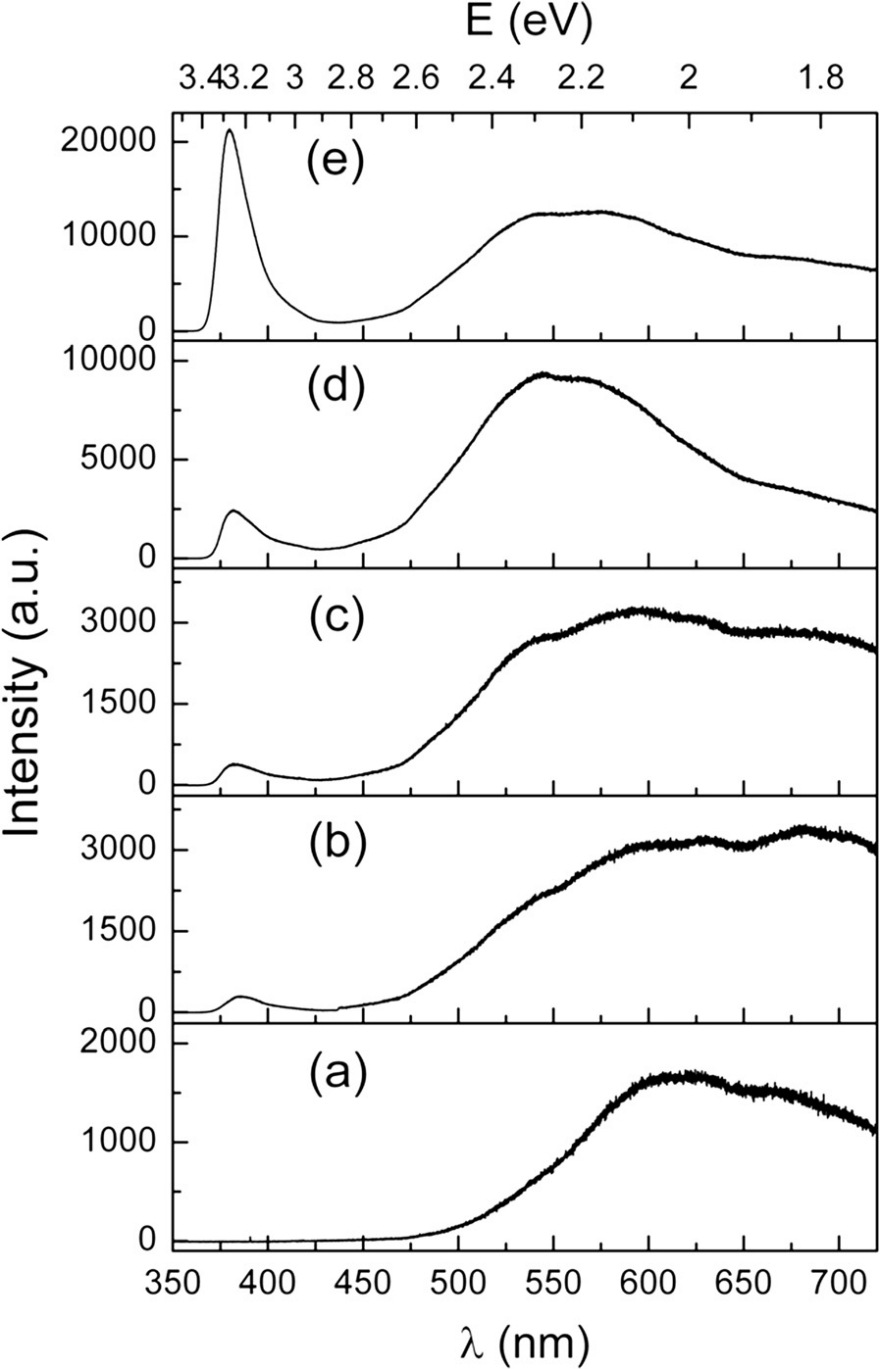
**PL analysis on the Si/ZnO heterostructure NWs.** PL spectra of (**a**) In/Si NWs and Si/ZnO core-shell NWs prepared at different ZnO growth times of (**b**) 0.5, (**c**) 1, (**d**) 1.5, and (**e**) 2 h.

The PL spectra of the In/Si NWs and ZnO nanostructures deposited on the In/Si NWs at different growth time are depicted in Figure 6. The In/Si NWs (Figure 6a) exhibit orange and red emissions with spectral range from 500 to 750 nm, centered at approximately 620 and 690 nm, respectively. The orange (approximately 620 nm) emission was caused by a defect emission due to incomplete oxidation on the surface of the In seeds [48], while the red (approximately 690 nm) emission is partially related to the quantum confinement effect in Si nanocrystallites surrounding the surface of the Si NWs [34,36]. Decorating the surface of the In/Si NWs with ZnO NPs creates a broader range of PL ranging from approximately 400 to 750 nm with an additional defect (green) emission from ZnO, centered at approximately 530 nm (Figure 6b). Meanwhile, a weak UV emission with a maximum reading at approximately 380 nm was also observed which is due to excitonic recombination corresponding to the near band edge emission of ZnO. Similar PL spectrum is observed for the ZnO NPs deposited at 1 h (Figure 6c) as well as traces of increment in the green and UV emissions. By increasing the ZnO growth time to 1.5 h, both the green and UV emissions were increased in relation to the suppression in the orange and red emissions. The suppression of the orange and red emissions from the In_2_O_3_ and nanocrystallites Si could be due to the full coverage of ZnO nanostructures on the In/Si NWs. Similarly, a change in the visible PL peak position from approximately 600 to 500 nm was also observed by Bera et al. [49] for the ZnS-coated ZnO NWs. This suggests that the visible emission can be changed by the formation of core-shell NWs. Further increase of the ZnO growth time to 2 h enhanced the UV emission and reduced the green emission of ZnO.

The green defect emission is normally observed for the ZnO nanostructures in addition to the near band edge emission. Although the origin of the green emission remains questionable, it is generally attributed to the transition of donor-acceptor pair related to the oxygen vacancies [14-16,50-52]. A number of studies suggested that the green emission is a surface-related process, where the radiative recombination of electrons with photogenerated holes trapped in singly ionized oxygen vacancies takes place in the surface structures of the ZnO [51,52]. In other words, this defect emission can be enhanced due to the large surface area of ZnO nanostructures under oxygen deficient conditions. Moreover, covering the surface of the ZnO nanostructures with surfactant or dielectric layers will eventually reduce or suppress the defect emission [53,54]. These findings correlate well with the results from our study. The high intensity of green to UV emission (approximately seven times) could be a feature of the defective states created by large quantities of ZnO NPs formed on the In/Si NWs. Only a minute increase in the green to UV peak intensity ratio was observed due to the volume expansion of the ZnO NPs by increasing the ZnO growth time from 0.5 to 1 h. The great increase in the surface area of ZnO by the hierarchical growth of ZnO NRs from the core-shell NWs resulted in the development of the green emission. Similar observation was reported by Wang et al. [52] in the comparison of PL properties of hierarchical grown ZnO NWs with ZnO NWs. Furthermore, our initial growth of ZnO NRs shows significant amount of kinking and bending structures. This indicates that there is a certain number of defect structures due to the nonstoichiometric (oxygen or zinc vacancies) ZnO which could be responsible for the defect emission.

Conversely, a reduction in the defect emission in conjunction with enhancement in the near band edge emission was also observed by further increasing the ZnO growth time to 2 h. The FESEM and TEM results showed the highly *c*-axis-oriented straight (no kinking) ZnO NRs growing from the core-shell NWs. The reduction of the defect emission can thus be explained by the improvement in the ZnO crystal lattices which minimizes the defect states of oxygen vacancies in ZnO. It is commonly known that the enhancement in the ZnO near band edge emission could be related to the size effect [55] and/or crystalline structure quality [50] of the ZnO NRs. Larger size of the ZnO NRs (diameter ≥70 nm) is always required to provide enough recombination center for the strong near band edge emission [55]. This is relevant to our case, where longer ZnO growth time increases the condensation of ZnO molecules, thus forming large sizes of ZnO NRs. According to our experiment, the branches of ZnO NRs with a diameter approximately 45 ± 13 nm and lengths of approximately 400 nm to 1 μm are sufficient for the enhancement in the near band edge emission. The UV emission peak of ZnO (centered at approximately 380 nm) was fitted using a Gaussian function to study the relation of PL peak width with the ZnO growth time. Full width at half maximum (FWHM) of the ZnO near band edge emission peak reduced from approximately 27 to 20 nm with the increase in ZnO growth time. The reduction in the FWHM value of the UV emission peak indicates an improvement in the crystalline structures of the ZnO NRs formed at longer growth time, which is consistent with the observations from XRD and HRTEM. This can be partly due to the annealing effect of the sample while increasing the ZnO growth time.

## Conclusions

The growth of ZnO nanostructures on In/Si NWs was studied using a vapor transport and condensation method. The results showed that a controllable morphology of ZnO nanostructures from ZnO NPs decorated to core-shell and hierarchical core-shell NWs can be achieved by controlling the condensation time of the ZnO vapors. The ZnO NRs which were hierarchically grown on the In/Si NWs were produced using In as a catalyst. XRD and HRTEM results indicated that the ZnO NPs had a tendency to be in (100) and (101) crystal planes, while the ZnO NRs on the Si/ZnO NWs advance along the [0001] direction. The Si/ZnO core-shell NWs revealed a broad range of PL at spectral range of 400 to 750 nm due to the combined emission of nanocrystallite Si, oxygen deficiency in In_2_O_3_ and oxygen-related defects in ZnO. Further, the growth of ZnO NRs from the core-shell NWs suppressed those defect emissions and enhanced the near band edge emission of ZnO.

## Competing interests

The authors declare that they have no competing interests.

## Authors' contributions

SK carried out the experimental parts on the sample preparation and characterization and drafted the manuscript. CF and SA participated in the statistical analysis and revised the manuscript. All authors read and approved the final manuscript.

## Supplementary Material

Additional file 1: Figure S1 Initial growth stage of ZnO NRs on In/Si NWs. (**a**) FESEM image and (**b**) TEM micrograph of the newly grown ZnO NRs. (**c**) High magnification TEM micrographs of In seed-capped ZnO NRs. **Figure S2.** HRTEM micrograph of the amorphous In2O3 and ZnO interface enlarged from a TEM micrograph of an In seed-capped ZnO NR. The TEM micrograph of the In seed-capped ZnO NR is inserted in the figure. Click here for file
