# Supplementary material for: Structural and photoluminescence studies on catalytic growth of silicon/zinc oxide heterostructure nanowires
Source: Nanoscale Res Lett. 2013 Apr 17;8(1):174. doi: 10.1186/1556-276X-8-174 (PMC3637626; doi:10.1186/1556-276X-8-174)
Supplement: Additional file 1: Figure S1 — Initial growth stage of ZnO NRs on In/Si NWs. (a) FESEM image and (b) TEM micrograph of the newly grown ZnO NRs. (c) High magnification TEM micrographs of In seed-capped ZnO NRs. Figure S2. HRTEM micrograph of the amorphous In2O3 and ZnO interface enlarged from a TEM micrograph of an In seed-capped ZnO NR. The TEM micrograph of the In seed-capped ZnO NR is inserted in the figure. [file 1556-276X-8-174-S1.pdf]

Supplementary Information for

Structural and photoluminescence studies on catalytic growth of silicon/zinc oxide heterostructure nanowires

Su Kong Chong[1,*], Chang Fu Dee[2], and Saadah Abdul Rahman[1]

[1]Low Dimensional Materials Research Centre, Department of Physics, University of Malaya, 50603 Kuala Lumpur, Malaysia. [2]Institute of Microengineering and Nanoelectronics (IMEN), Universiti Kebangsaan Malaysia (UKM), Bangi, Selangor, Malaysia.

[*] Corresponding author. Tel.:+603-79674147, Email address: sukong1985@yahoo.com.my

---

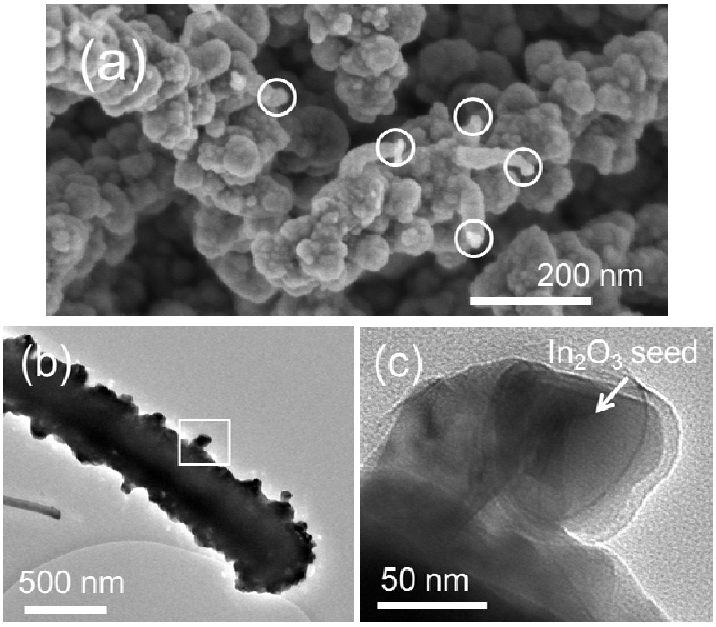

Figure S1 Initial growth stage of ZnO NRs on In/Si NWs. (a) FESEM image and (b) TEM micrograph of the newly grown ZnO NRs. (c) High magnification TEM micrographs of In seed-capped ZnO NRs.

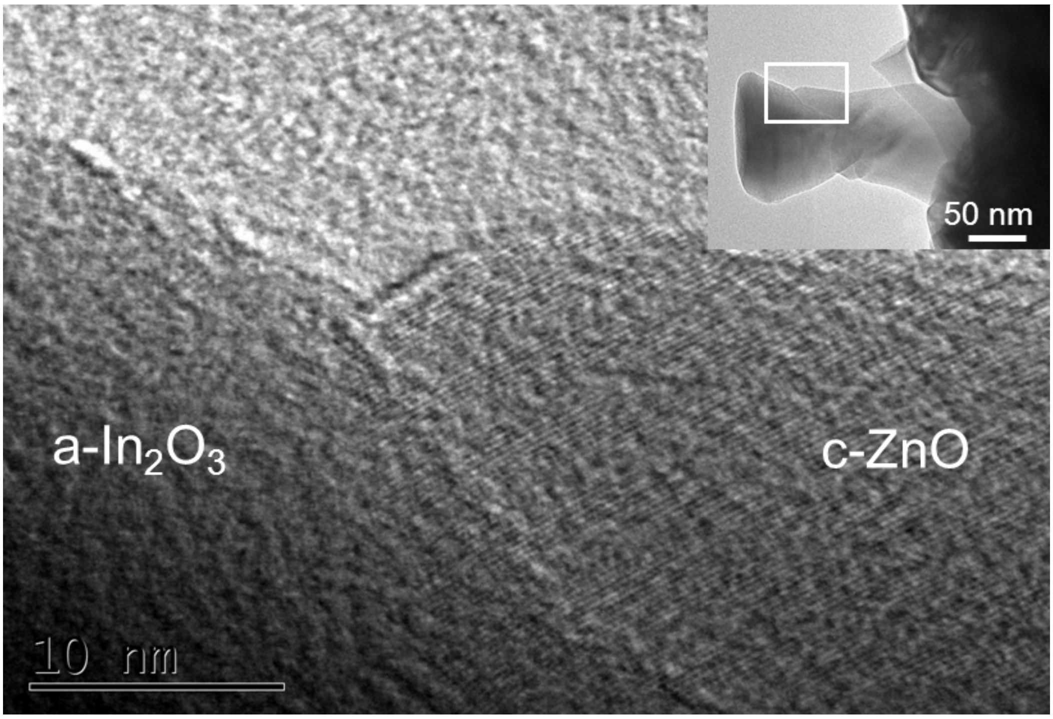

Figure S2 HRTEM micrograph of the amorphous In_2O_3 and ZnO interface enlarged from a TEM micrograph of an In seed-capped ZnO NR. The TEM micrograph of the In seed-capped ZnO NR is inserted in the figure.
